# Identification of Postpartum Depression in Electronic Health Records: Validation in a Large Integrated Health Care System

**DOI:** 10.2196/43005

**Published:** 2023-03-01

**Authors:** Jeff Slezak, David Sacks, Vicki Chiu, Chantal Avila, Nehaa Khadka, Jiu-Chiuan Chen, Jun Wu, Darios Getahun

**Affiliations:** 1 Kaiser Permanente Southern California Pasadena, CA United States; 2 Keck School of Medicine University of Southern California Los Angeles, CA United States; 3 Program in Public Health Susan and Henry Samueli College of Health Sciences University of California Irvine, CA United States; 4 Kaiser Permanente Bernard J. Tyson School of Medicine Pasadena, CA United States

**Keywords:** validation, postpartum depression, electronic health records, pregnancy, health care system, diagnosis codes, pharmacy records, health data, data collection, implementation, eHealth record, depression, mental well-being, women's health

## Abstract

**Background:**

The accuracy of electronic health records (EHRs) for identifying postpartum depression (PPD) is not well studied.

**Objective:**

This study aims to evaluate the accuracy of PPD reporting in EHRs and compare the quality of PPD data collected before and after the implementation of the *International Classification of Diseases, Tenth Revision* (*ICD-10*) coding in the health care system.

**Methods:**

Information on PPD was extracted from a random sample of 400 eligible Kaiser Permanente Southern California patients’ EHRs. Clinical diagnosis codes and pharmacy records were abstracted for two time periods: January 1, 2012, through December 31, 2014 (*International Classification of Diseases, Ninth Revision* [*ICD-9*] period), and January 1, 2017, through December 31, 2019 (*ICD-10* period). Manual chart reviews of clinical records for PPD were considered the gold standard and were compared with corresponding electronically coded diagnosis and pharmacy records using sensitivity, specificity, positive predictive value (PPV), and negative predictive value (NPV). Kappa statistic was calculated to measure agreement.

**Results:**

Overall agreement between the identification of depression using combined diagnosis codes and pharmacy records with that of medical record review was strong (κ=0.85, sensitivity 98.3%, specificity 83.3%, PPV 93.7%, NPV 95.0%). Using only diagnosis codes resulted in much lower sensitivity (65.4%) and NPV (50.5%) but good specificity (88.6%) and PPV (93.5%). Separately, examining agreement between chart review and electronic coding among diagnosis codes and pharmacy records showed sensitivity, specificity, and NPV higher with prescription use records than with clinical diagnosis coding for PPD, 96.5% versus 72.0%, 96.5% versus 65.0%, and 96.5% versus 65.0%, respectively. There was no notable difference in agreement between *ICD-9* (overall κ=0.86) and *ICD-10* (overall κ=0.83) coding periods.

**Conclusions:**

PPD is not reliably captured in the clinical diagnosis coding of EHRs. The accuracy of PPD identification can be improved by supplementing clinical diagnosis with pharmacy use records. The completeness of PPD data remained unchanged after the implementation of the *ICD-10* diagnosis coding.

## Introduction

Postpartum depression (PPD), major or minor depressive episodes occurring within 12 months after childbirth, is a common obstetric complication in the United States, with a prevalence of 13.2% in 2018 [1]. The American College of Obstetricians and Gynecologists recommends all obstetrics care providers conduct comprehensive screening for PPD and anxiety disorders using a validated instrument for each patient separately during their postpartum visit [2]. Meanwhile, the American Academy of Pediatrics recommended routine PPD screening to be integrated at well-child visits (1-, 2-, 4-, and 6-month infant visits) [3]. The US Preventive Services Task Force also supports the provision of depression screening during postpartum visits, citing moderate net benefits for identifying those affected and recommending referrals to counseling interventions [4]. It is important to identify those with PPD because undetected or untreated depressive episodes can negatively impact the patient and their infant’s health and well-being. For instance, about 9% of pregnancy-related deaths were due to mental health conditions [5]. Early PPD was also associated with increased behavior disturbances in the infant [6]. Moreover, other potential risk factors, including a prior history of depression, depression and anxiety episodes during pregnancy, preterm birth and lower infant birth weight, traumatic birth experience, stressful life events during early postpartum, and low social support, have been linked with PPD [7-9].

Health systems previously used the *International Classification of Diseases, Ninth Revision* (*ICD-9*), an official coding system to identify hospital-related diagnoses and procedures in the United States [10]. However, the Kaiser Permanente health systems shifted to using the *International Classification of Diseases, Tenth Revision* (*ICD-10*) codes after October 1, 2015, which has significant improvements over *ICD-9* for many clinical codes [11]. However, Stewart et al [12] concluded that there is a need to perform a validation of diagnosis codes for each mental health condition following the *ICD-10* transition. Colvin et al [13] used a data linkage of national pharmacy records and hospital admission information to identify patients with major depressive episodes in pregnancy but found the use of either source alone to be inadequate.

While there are multiple validated scales to screen for PPD, like the Patient Health Questionnaire (9-item) and the Edinburgh Postnatal Depression Scale, validation of these measures has been performed using *ICD-9* or *ICD-10* diagnostic codes as the gold standard [14,15]. Several studies have also developed machine learning algorithms using electronic health record (EHR) data to create risk-based models and examined whether they can predict PPD in large health care systems, relying on PPD ascertained using *ICD-9* or *ICD-10* codes [16,17]. However, the accuracy of *ICD-9* and *ICD-10* codes as the gold standard in ascertaining PPD has not been established previously. Prior validation of *ICD-9* and *ICD-10* found high positive predictive values (PPVs) for ascertaining general depression (89.7% and 89.5%, respectively), but these were not specific to the postpartum period [18]. This study aimed to assess the validity of ascertaining PPD diagnosis using the EHR from a large integrated health care delivery system, Kaiser Permanente Southern California (KPSC).

## Methods

### Cohort Selection

We identified a random sample of 400 women with live birth records in the Air Pollution and Pregnancy Complications in Complex Urban Environments (APPCUE) study [19] between January 1, 2008, and December 31, 2018, within KPSC, a large integrated health system. The APPCUE study was a retrospective cohort study conducted in collaboration between KPSC and the University of California, Irvine with access to KPSC’s comprehensive EHRs. The APPCUE study included all singleton births at KPSC facilities. The EHRs contain patient-level data from out- and inpatient clinical care, including *ICD-9, Clinical Modification* or *ICD-10, Clinical Modification* diagnosis and procedure codes, as well as pharmacy and laboratory test records. From 236,759 pregnancies during the study period, we excluded pregnancies resulting in nonlive births (n=8422) and patients who were not members from the start of their pregnancy through a 1-year postpartum period (n=70,836) to have a complete medical history for this validation study. Of the remaining 157,501 pregnancies, we selected a random sample of 400. Simple random sampling was used to select 100 patients from groups based on EHR data: those without any diagnostic or pharmacy use record for PPD, those with only a diagnostic code for PPD, those with only a pharmacy record indicating treatment for PPD, and those with both diagnostic and pharmacy indications. Additionally, each sample was evenly split (50 each) between the *ICD-9* diagnosis code era (date of delivery 2012-2014) and the *ICD-10* era (2017-2019).

### Outcomes

EHR outcomes were determined by the presence of PPD diagnosis codes in inpatient or outpatient encounters in the 12 months after delivery, new prescription order, or pharmacy dispense for the treatment of PPD. Diagnosis codes during the *ICD-9* coding period were 300.4, 309.0, and 311 and during the *ICD-10* period were F32.9, F33.0, F33.2, F33.3, F33.41, F33.9, F34.1, F43.21, and F53.0. Medications included were bupropion, Celexa, citalopram, Cymbalta, desvenlafaxine, duloxetine, Effexor, escitalopram, fluoxetine, Lexapro, paroxetine, Paxil, Pristiq, Prozac, sertraline, venlafaxine, Wellbutrin, and Zoloft.

Gold standard PPD outcomes were determined by review of health records by trained research personnel, who documented any diagnosis or finding of PPD in the record, including in free-text encounter notes, as well as any prescription given for the treatment of PPD. These included new prescriptions for the treatment of PPD. PPD diagnosis and medication were documented independently, both for the EHR data and the chart review. A mother was considered to have PPD if she had either a diagnosis or a prescription noted in the EHR within 1 year postpartum.

### Quality Assurance

Multiple individuals were trained on reviewing charts, and a double chart review was performed at the beginning of data collection as a training exercise and near the middle and at the end of data collection to verify data quality and consistency. At each point, eight charts were randomly selected for review by two abstractors. In case of disagreement on the findings, abstractors met with the trainer to determine the correct result.

### Statistical Analysis

The patient population was described in terms of demographics, smoking status, prenatal care, and birth weight using percentages. These characteristics were also described for the study population of the APPCUE study [19] and all live births among KPSC members and the state of California during the study timeframe. The chi-square test was used to compare the distribution of characteristics in the study sample to the APPCUE population, all KPSC births, and the California birth cohort.

Manual chart review findings were treated as the true PPD status. The sensitivity, specificity, PPV, and negative predictive value (NPV) of the electronic records to identify true PPD status were calculated and presented as a percentage and 95% exact binomial CI. Agreement between electronic records and manual review was calculated using the kappa statistic, which adjusts for agreement expected due to random chance, and its 95% CI. The area under the receiver operating characteristic curve was calculated. Each measure was calculated overall and within the *ICD-9* and *ICD-10* coding eras separately. There was no missing data for PPD status; those without documented PPD diagnosis or medication were taken to not have PPD. For patient characteristics, a missing category was included when presenting the data.

The primary analysis focused on the ability of EHRs to capture PPD, while secondary analyses examined the agreement of diagnosis and prescription records separately. The sample size was selected so that the expected width of the CIs for sensitivity and PPV would be at most 10% for the full sample and 13% for the *ICD-9* and *ICD-10* periods if the true sensitivity and PPV were 80%. Higher sensitivity and PPV would yield narrower CIs. The STARD (Standards for Reporting Diagnostic Accuracy Studies) guidelines were followed. All analyses were performed in SAS version 9.4 (SAS Institute).

### Ethics Approval

The study was approved by the institutional review board of KPSC and received a waiver for informed consent (IRB 12110).

## Results

### Cohort Selection

Table 1 shows the distribution of the APPCUE study cohort as well as the overall KPSC birth cohort during the study period. Nearly half (194/400, 48.5%) were Latina, most (379/400, 94.8%) received prenatal care starting in the first trimester, and most (354/400, 88.5%) delivered at 37 weeks of gestation or later. The study sample generally has very similar characteristics to the APPCUE study cohort overall and all KPSC births during the period, though there are some differences relative to all deliveries in the state of California, notably a higher percentage of non-Hispanic White mothers (113/400, 28.3% vs 372,037/2,874,396, 12.9%), older mothers (259/400, 64.8% age ≥30 years vs 1,465,998/2,874,396, 50.0%), and generally higher educational attainment (199/400, 49.8% with at least a college degree vs 1,047,594/2,874,396, 36.5%).

**Table 1:** Characteristics of the study sample and women delivered in all Kaiser Permanente Southern California (KPSC) hospitals and the state of California (2012-2014 and 2017-2019).

Characteristics			Chart review sample^a^ (N=400), n (%)		APPCUE^b^ study population (N=157,501), n (%)		*P* value		All KPSC births (N=236,759), n (%)		*P* value		All California State births^c^ (N=2,874,396), n (%)		*P* value
**Maternal age (years)**							.42				.01				<.001
	<20	10 (2.5)		4665 (3.0)				6804 (3.0)				144,945 (5.0)			
	20-29	131 (32.8)		56,679 (36.0)				92,203 (40.4)				1,263,453 (44.0)			
	30-34	153 (38.3)		54,810 (34.8)				75,633 (33.1)				843,010 (29.3)			
	≥35	106 (26.5)		41,347 (26.3)				53,697 (23.5)				622,988 (21.7)			
**Race/ethnicity**							.31				.29				<.001
	Non-Hispanic White	113 (28.3)		39,219 (24.9)				55,218 (24.2)				372,037 (12.9)			
	Non-Hispanic Black	32 (8.0)		10,862 (6.9)				16,207 (7.1)				68,195 (2.4)			
	Hispanic	194 (48.5)		78,853 (50.1)				117,162 (51.3)				1,356,354 (47.2)			
	Asian/Pacific Islander	47 (11.8)		22,783 (14.5)				31,318 (13.7)				213,499 (7.4)			
	Others/unknown	14 (3.5)		5784 (3.7)				8432 (3.7)				864,311 (30.1)			
**Educational attainment**							.36				.20				<.001
	Less than high school	9 (2.2)		4355 (2.8)				6925 (3.0)				435,360 (15.1)			
	High school graduate	83 (20.8)		35,411 (22.5)				55,598 (24.4)				694,118 (24.1)			
	Some college	99 (24.8)		32,616 (20.7)				50,153 (22.0)				558,288 (19.4)			
	Bachelor’s/associate’s degree	126 (31.5)		54,293 (34.5)				75,849 (33.2)				729,896 (25.4)			
	Master’s degree/above	73 (18.3)		27,388 (17.4)				34,556 (15.1)				317,698 (11.1)			
	Missing	10 (2.5)		3438 (2.2)				5256 (2.3)				139,036 (4.8)			
**Household income (US $)**							.64				.25				—^d^
	<30,000	16 (4.0)		5194 (3.3)				8318 (3.6)				—			
	30,000-49,999	90 (22.5)		39,969 (25.4)				61,562 (27.0)				—			
	50,000-69,999	124 (31.0)		47,864 (30.4)				69,844 (30.6)				—			
	70,000-89,999	82 (20.5)		32,486 (20.6)				45,469 (19.9)				—			
	≥90,000	88 (22.0)		31,925 (20.3)				42,782 (18.7)				—			
**Prenatal care initiation**							.52				<.001				<.001
	First trimester	379 (94.8)		147,017 (93.3)				199,866 (87.5)				2,386,232 (83.0)			
	No or late care	20 (5.0)		9860 (6.3)				26,966 (11.8)				442,493 (15.4)			
	Missing	1 (0.2)		624 (0.4)				1505 (0.7)				45,671 (1.6)			
Smoking during pregnancy			23 (5.8)		6420 (4.1)		.09		10,256 (4.5)		.16		46,977 (1.6)		<.001
**Gestational age (weeks)**							.13				.08				.16
	<34	14 (3.5)		3412 (2.2)				4779 (2.1)				66,099 (2.3)			
	34-36	32 (8.0)		9865 (6.3)				13,933 (6.1)				180,352 (6.3)			
	≥37	354 (88.5)		144,192 (91.5)				209,553 (91.8)				2,624,620 (91.3)			
	Missing	0 (0.0)		32 (0.0)				72 (0.0)				3325 (0.1)			

^a^Sample is based on data from KPSC electronic health records 2012-2014 and 2017-2019.

^b^APPCUE: Air Pollution and Pregnancy Complications in Complex Urban Environments.

^c^Data from the natality information of the Center for Disease Control and Prevention [20].

^d^Data not available.

### Outcomes

The overall agreement of EHR-identified PPD (based on either a diagnosis or a prescription) with medical record review was high, with a kappa of 84.7% (95% CI 78.8%-90.6%). The EHR identified 281 of 286 cases (sensitivity 98.3%, 95% CI 96.0%-99.4%) while maintaining high specificity (95.0%, 95% CI 88.7%-98.4%), PPV (93.7%, 95% CI 90.3%-96.1%), and NPV (95.0%, 95% CI 88.7%-98.4%). There was little difference in the overall agreement between the *ICD-9* coding era (κ=86.0%, 95% CI 78.0%-94.0%) and the *ICD-10* era (κ=83.4%, 95% CI 74.8%-92.1%; Table 2).

**Table 2:** Identification of postpartum depression using diagnostic codes and/or pharmacy records–based data sources before and after implementation of the ICD-10 code in the Kaiser Permanente Southern California system in 2015 (N=400).

		TP^a^, n	TN^b^, n	FP^c^, n	FN^d^, n	Sensitivity, % (95% CI)	Specificity, % (95% CI)	PPV^e^, % (95% CI)	NPV^f^, % (95% CI)	Kappa (95% CI)	AUC^g^
**Combined electronic diagnosis codes and pharmacy records**											
	Overall	281	95	19	5	98.3 (96.0-99.4)	83.3 (75.2-89.7)	93.7 (90.3-96.1)	95.0 (88.7-98.4)	0.85 (0.79-0.91)	0.91
	2012-2014	141	48	9	2	98.6 (95.0-99.8)	84.2 (72.1-92.5)	94.0 (88.9-97.2)	96.0 (86.3-99.5)	0.86 (0.78-0.94)	0.91
	2017-2019	140	47	10	3	97.9 (94.0-99.6)	82.5 (70.1-91.3)	93.3 (88.1-96.8)	94.0 (83.5-98.7)	0.83 (0.75-0.92)	0.90
***ICD-9*^h^/*ICD-10*^i^ diagnosis codes only**											
	Overall	187	101	13	99	65.4 (59.6-70.9)	88.6 (81.3-93.8)	93.5 (89.1-96.5)	50.5 (43.4-57.6)	0.44 (0.36-0.52)	0.77
	2012-2014	94	51	6	49	65.7 (57.3-73.5)	89.5 (78.5-96.0)	94.0 (87.4-97.8)	51.0 (40.8-61.1)	0.45 (0.34-0.56)	0.78
	2017-2019	93	50	7	50	65.0 (56.6-72.8)	87.7 (76.3-94.9)	93.0 (86.1-97.1)	50.0 (39.8-60.2)	0.43 (0.32-0.54)	0.76
**Pharmacy records only**											
	Overall	194	108	6	92	67.8 (62.1-73.2)	94.7 (88.9-98.0)	97.0 (93.6-98.9)	54.0 (46.8-61.1)	0.51 (0.43-0.59)	0.81
	2012-2014	97	54	3	46	67.8 (59.5-75.4)	94.7 (85.4-98.9)	97.0 (91.5-99.4)	54.0 (43.7-64.0)	0.51 (0.40-0.62)	0.81
	2017-2019	97	54	3	46	67.8 (59.5-75.4)	94.7 (85.4-98.9)	97.0 (91.5-99.4)	54.0 (43.7-64.0)	0.51 (0.40-0.62)	0.81

^a^TP: true positive.

^b^TN: true negative.

^c^FP: false positive.

^d^FN: false negative.

^e^PPV: positive predictive value.

^f^NPV: negative predictive value.

^g^AUC: area under the receiver operating characteristic curve.

^h^*ICD-9: International Classification of Diseases, Ninth Revision*.

^i^*ICD-10: International Classification of Diseases, Tenth Revision*.

Electronic diagnosis records alone were not able to accurately identify PPD, only identifying 187 of 286 cases (sensitivity 65.4%, 95% CI 59.6%-70.9%), with low NPV (50.5%, 95% CI 43.4%-57.6%). PPV (93.5%, 95% CI 89.1%-96.5%) and specificity (88.6%, 95% CI 81.3%-93.8%) were high, however (Table 2). Results were similar when using EHR prescription records alone (sensitivity 67.8%, 95% CI 62.1%-73.2%; specificity 94.7%, 95% CI 88.9%-98.0%; PPV 97.0%, 95% CI 93.6%-98.9%; NPV 54.0%, 95% CI 46.8%-61.1%).

Considering only medication data, the reliability of EHR data for identifying prescriptions for PPD was high, with an overall kappa of 92.5% (95% CI 88.8%-96.2%). Agreement was very high in both the *ICD-9* (κ=92.0%, 95% CI 86.6%-97.4%) and *ICD-10* eras (κ=93.0%, 95% CI 87.9%-98.1%; Table 3). Sensitivity, specificity, PPV, and NPV were all at or above 96% (Table 3).

Agreement for *ICD* diagnostic codes between EHR and manual chart review was much lower overall (κ=55.0%, 95% CI 47.1%-62.9%; Table 3). The PPV was high (90.0%, 95% CI 85.0%-93.8%), with sensitivity lower (72.0%, 95% CI 66.0%-77.5%) and specificity and NPV much lower (both 65.0%, 95% CI 58.0%-71.6%; Table 3). Agreement was similar between the *ICD-9* (κ=58.0%, 95% CI 47.1%-68.9%) and *ICD-10* (κ=52.0%, 95% CI 40.5%-63.5%) eras (Table 3).

**Table 3:** Identification of postpartum depression based on individual data sources before and after implementation of the ICD-10 code in the Kaiser Permanente Southern California system in 2015 (N=400).

			TP^a^, n	^b^TN, n		FP^c^, n		FN^d^, n		Sensitivity, % (95% CI)	Specificity, % (95% CI)		PPV^e^, % (95% CI)	NPV^f^, % (95% CI)		Kappa (95% CI)		AUC^g^
***ICD-9*^h^/*ICD-10*^i^ diagnosis codes only**																		
	Overall	180		130	20		70		72.0 (66.0-77.5)		86.7 (80.2-91.7)	90.0 (85.0-93.8)		65.0 (58.0-71.6)	0.55 (0.47-0.63)		0.79	
	2012-2014	92		66	8		34		73.0 (64.4-80.5)		89.2 (79.8-95.2)	92.0 (84.8-96.5)		66.0 (55.8-75.2)	0.58 (0.47-0.69)		0.78	
	2017-2019	88		64	12		36		71.0 (62.1-78.8)		84.2 (74.0-91.6)	88.0 (80.0-93.6)		64.0 (53.8-73.4)	0.52 (0.41-0.64)		0.81	
**Pharmacy records only**																		
	Overall	192		193	8		7		96.5 (92.9-98.6)		96.0 (92.3-98.3)	96.0 (92.3-98.3)		96.5 (92.9-98.6)	0.93 (0.89-0.96)		0.96	
	2012-2014	96		96	4		4		96.0 (90.1-98.9)		96.0 (90.1-98.9)	96.0 (90.1-98.9)		96.0 (90.1-98.9)	0.92 (0.87-0.97)		0.97	
	2017-2019	96		97	4		3		97.0 (91.4-99.4)		96.0 (90.2-98.9)	96.0 (90.1-98.9)		97.0 (91.5-99.4)	0.93 (0.88-0.98)		0.96	

^a^TP: true positive.

^b^TN: true negative.

^c^FP: false positive.

^d^FN: false negative.

^e^PPV: positive predictive value.

^f^NPV: negative predictive value.

^g^AUC: area under the receiver operating characteristic curve.

^h^*ICD-9*: *International Classification of Diseases, Ninth Revision*.

^i^*ICD-10*: *International Classification of Diseases, Tenth Revision*.

### Quality Assurance

During the training process, 8 charts were independently reviewed by two chart abstractors. Their assessments of medication use for PPD agreed for all 8 records (100%), while the assessment of a diagnostic finding agreed for 7 (88%). After training was complete, another 8 records were independently reviewed. All 8 (100%) agreed in their findings for both medications and diagnoses.

## Discussion

### Principal Findings

This validation study demonstrated the potential to improve the accuracy of PPD case identification from an EHR when using diagnosis codes in conjunction with pharmacy records. The combination of clinical codes and prescription pharmacy records yielded much greater sensitivity and NPV, with no notable loss in specificity or PPV, compared with using either the diagnosis codes or pharmacy records alone. Using either record alone would result in significant undercounting, each missing about one-third of those with PPD, compared to the 95% identified using both together. Furthermore, we observed no significant difference in the *ICD-9* and *ICD-10* codes in terms of ascertaining PPD cases.

We found that electronic records of PPD diagnosis were not a reliable indicator of PPD diagnostic findings identified through chart review, relative to pharmacy records. Pharmacy records have both a sensitivity and specificity much higher than that seen for diagnosis codes.

The quality of data extracted from EHRs for pharmacoepidemiologic research has been proven to be valuable. Although using clinical diagnosis codes for perinatal epidemiology studies has limitations, the use of KPSC’s comprehensive pharmacy use records enhances the identification of PPD cases (sensitivity 98.3%, specificity 95.0%, PPV 93.7%, and NPV 95.0%).

While switching from *ICD-9* to *ICD-10* coding created some complexity, we did not see a significant difference in the accuracy of the electronic diagnosis records between the *ICD-9* and *ICD-10* coding eras. This is reassuring, as studies would not need to be limited to one era or the other for the sake of accuracy. Additionally, the prevalence of PPD identified in both periods is essentially the same, suggesting that both *ICD-9* and *ICD-10* coding systems identify patients with PPD at the same rate, negating any need to adjust prevalence estimates to account for the difference.

Accurate characterization of those with PPD is crucial to performing valid research on this condition. Many researchers rely on electronic records due to a lack of access to detailed patient histories or a lack of time to review these records. Our study suggests that researchers can accurately identify PPD from EHRs using both diagnosis and pharmacy records.

### Comparison to Prior Work

Prior research validating diagnosis codes for identifying general depression found the PPV to be similar to that seen in our study (89.7% for *ICD-9* and 89.5% for *ICD-10*), but these were not specific to the postpartum period [18]. These findings highlight the continuing debate regarding the use of diagnosis codes alone for epidemiological studies. Our study concurs with prior findings that the sensitivity and specificity of case ascertainment can be improved by concurrently using both diagnosis and pharmacy records [13]. Therefore, researchers should not rely exclusively on either diagnostic codes or pharmacy records for PPD case ascertainment.

### Strengths and Limitations

There are some potential limitations to this study. First, while the KPSC EHR is comprehensive, it may not capture care received outside the system if it is not submitted for reimbursement. Specifically, members may receive mental health counseling from non-KPSC providers, and a PPD diagnosis made in that setting may not be entered into the KPSC medical record, resulting in a potentially missed PPD diagnosis and an underestimate of the sensitivity of diagnosis coding. However, these diagnoses may still be identified during regular clinical care within KPSC, hence limiting the number of potentially missed diagnoses.

Second, misclassification is also possible as variables were ascertained from clinical diagnosis codes and pharmacy record notes. In addition, there is the potential for misclassification of PPD within the data sources if women are unaware of the condition, do not seek medical care, or the diagnosis or treatment is not recorded in the clinical notes. Any completely undocumented cases would result in an underestimate of PPD in the population, though its potential effect on our validation is unknown. Finally, due to the small number of records reviewed in some groups, we were not able to look for differences in medical record accuracy within subsets of the population, including by age and race/ethnicity. If differences are present, this will limit the generalizability of these findings to other populations with different demographics.

Strengths of this study include the comprehensive medical record and chart review conducted to identify PPD in this patient population. The training and validation of the chart review process helped to ensure that the gold standard PPD identification was accurate.

### Conclusions

This validation study of PPD that was carried out in a large integrated health care system in Southern California has demonstrated that PPD data ascertainment based on a combination of diagnosis codes and prescription medication records from the EHR is highly accurate for pharmacoepidemiologic studies. Neither diagnosis codes alone nor prescription records alone are sufficient to capture PPD cases.

## Abbreviations

APPCUE: Air Pollution and Pregnancy Complications in Complex Urban Environments
EHR: electronic health record
ICD-9: International Classification of Diseases, Ninth Revision
ICD-10: International Classification of Diseases, Tenth Revision
KPSC: Kaiser Permanente Southern California
NPV: negative predictive value
PPD: postpartum depression
PPV: positive predictive value
STARD: Standards for Reporting Diagnostic Accuracy Studies

## Data Availability

Most of the data that support the findings of this study are available on request from the corresponding author. The complete data set is not publicly available due to privacy, institutional approval, and/or ethical restrictions. Study data come from patient electronic health records and birth certificates from the state of California. Data from patient health records cannot be shared without signed confidentiality agreements. Some of the data that support the findings of this study are available from the state of California. Restrictions apply to the availability of these data, which were used under license and approval for this study. Data can be made available by the authors provided that all required approvals are obtained from the departments in the state that oversees the use of state vital records data. To obtain California birth certificate data, researchers can email cphs@chhs.ca.gov or visit their website [21]. Requests for data may be sent to JS (Jeff.M.Slezak@kp.org) and DG (Darios.T.Getahun@kp.org).
